# KRAS Mutations in Colorectal Cancer: Relationship With Clinicopathological Characteristics and Impact on Clinical Outcomes in Saudi Arabia

**DOI:** 10.7759/cureus.23656

**Published:** 2022-03-30

**Authors:** Mohammed Alghamdi, Nujud Alabdullatif, Ajeed Al-Rashoud, Joud Alotaibi, Nouf Alhussaini, Sedra Elsirawani, Haneen Somily, Norah Alkhudair, Maram AlOtaiby, Shaik S Ahamed, Nashwa Abd El-Aziz

**Affiliations:** 1 Department of Medical Oncology, King Saud University Medical City, King Saud University, Riyadh, SAU; 2 Department of Clinical Pharmacy, College of Pharmacy, King Saud University, Riyadh, SAU; 3 Molecular Genetics Pathology Unit, Department of Pathology, King Saud University, Riyadh, SAU; 4 Department of Family and Community Medicine, College of Medicine, King Saud University, Riyadh, SAU; 5 Department of Medical Oncology, South Egypt Cancer Institute, Assiut University, Assiut, EGY

**Keywords:** survival, prognosis, clinical, kras, saudi arabia, colorectal cancer

## Abstract

Background

Few studies have addressed the prevalence and prognostic impacts of *KRAS* mutations in Saudi patients with colorectal cancer (CRC). The present study aimed to address the prevalence of *KRAS *mutations and evaluate their impact on clinical outcomes (if any) among Saudi patients.

Methods

This retrospective cohort study was conducted at King Saud University Medical Centre (KSUMC), Saudi Arabia. All medical records of biopsy-proven CRC patients between 2015 and 2021 were reviewed. Statistical analysis was carried out to address the associations between* KRAS* mutations and the clinicopathological patients’ variables and survival.

Results

*KRAS* mutations were found in 97/194 (50%) CRC patients. In comparison to wild type *KRAS *tumors, *KRAS- *mutated ones had shown a trend toward right-sided tumors (30% and 4.3% vs 16% and 1.1%, p-value = 0.032, respectively) and peritoneal metastases (34% vs 19%, p-value = 0.014). Older age at diagnosis, gender, tumor grade, microsatellite instability (MSI), tumor stage (T), and the presence of distant metastasis were independent prognostic factors for poor overall survival (OS). There was no significant association between *KRAS *mutations and the hazard of mortality (HR: 0.653, 95% CI 0.873-1.134, p = 0.131). For progression-free survival (PFS), older age at presentation, MSI, tumor nodal stage (N), the presence of liver and lung metastasis, and recurrence were poor prognostic factors for PFS. There was no significant relation between *KRAS *mutations and PFS (HR ratio: 0.756, 95% CI 0.229-2.497, p = 0.646).

Conclusions

The prevalence of KRAS mutations in CRC patients was similar to that observed in previous studies of Saudi patients. *KRAS *mutations showed a trend toward right-sided tumors and peritoneal metastases. Survival was significantly related to different clinicopathologic variables of the study cohort but was not affected by the *KRAS* mutational status.

## Introduction

The Cancer Registry in Saudi Arabia has shown that colorectal cancer (CRC) is common, being the most commonly diagnosed cancer among males while it comes as the third commonest among females [1]. Relevant studies had shown that CRC is a heterogeneous disease that arises from multiple genetic and cellular alterations. Scientists’ efforts aimed at identifying molecular phenotypes for CRC are crucial to the patients’ management, as they can predict tumor response to treatment and guide molecular-targeted therapies [2-3].

Kirsten-ras (KRAS) is an oncogene that is reported to be activated through mutations in 30% to 50% of patients with CRC [4]. Characteristically, almost all of these KRAS mutations occur in codons 12 or 13 and rarely in codon 61 [5-6].

Despite the fact of the intimate relation of KRAS mutations to the responses to treatment in patients with CRC, the effect of KRAS on prognosis is still debatable [7-10]. While many reports have advocated that KRAS is a negative prognostic marker, others have observed no prognostic significance, and few studies have addressed the prevalence and prognostic impacts of KRAS mutations in Saudi patients with CRC [7-11]. Therefore, the present study aimed to address the prevalence of KRAS mutations and evaluate their impact on clinical outcomes (if any) among Saudi patients seeking advice at a tertiary oncology center.

## Materials and methods

Study design and population

This retrospective cohort study was conducted at the Oncology Centre, King Saud University Medical Centre (KSUMC), King Saud University, Riyadh, Saudi Arabia. We retrospectively reviewed all medical records of biopsy-proven colorectal cancer (CRC) patients who were admitted to the hospital between 2015 and 2021 and underwent surgery or were candidates for chemotherapy, radiotherapy, or both (n = 194). There were no exclusion criteria. A non-probability consecutive sampling technique was used for all patients who met the inclusion criteria.

The research group members, using patients’ medical records, collected data regarding variables such as demographic characteristics (age and gender) and pathological tumor features (histopathological type, primary site, grade of differentiation, and staging). The overall survival (OS) was calculated as the interval between the date of diagnosis and death or last follow-up, and progression-free survival (PFS) was calculated as the interval between the initiation of treatment and disease progression or death due to any cause or last follow-up.

DNA isolation and analysis of *KRAS* mutations

Five to 10 μm-thick sections from the patient's formalin-fixed, paraffin-embedded tissue samples were used for DNA isolation. Malignant cells then were lysed in order to extract genomic DNA and perform real-time PCR amplification. A *KRAS* mutations test, Biocartis Idylla^TM^ (Mechelen, Belgium), was utilized to detect the presence of 21 *KRAS *mutations in exons 2,3, and 4.

Ethical considerations

Approval for this study was obtained from the Institutional review board (IRB) at King Saud University (IRB No.: E-20-5374). All participants received a written consent form upon opening a medical file at KSUMC. Confidentiality and anonymity were maintained throughout the study.

Statistical analysis

Categorical data were expressed using frequencies and percentages. Numerical data were described as the mean and standard deviation or the median and interquartile range, as appropriate. The chi-squared test was used to assess the association between *KRAS* mutations and other clinical variables. A Kaplan Meier analysis was carried out to compare the mean survival time between patients with *KRAS*-mutated and wild-type CRCs. A Cox regression analysis was carried out and stratified by patients’ gender and age, tumor stage, and mutational status, and hazard ratios (HR) were calculated. Data were analyzed using SPSS version 26.0 statistical software (IBM Corp., Armonk, NY). A p-value of <0.05 was considered statistically significant.

## Results

Patients and tumor characteristics

The study included 194 patients. Gender differences were observed, where males and females represented 53.3% and 46.7%, respectively. The mean age at diagnosis was 58 ± 13 years with 77% of patients diagnosed above the age of 50 years. Approximately 44% of CRC patients had a complete or partial response to treatment, and 29% had disease progression or metastasis. The most common primary tumor site was the left colon (38%), followed by the rectum (31%) and the right colon (22%). Histologically, 93% of CRCs were adenocarcinomas in origin. Seventy-one percent of tumors had a low grade of differentiation while 16% were highly differentiated.

Associations between *KRAS* mutations and clinicopathological variables

*KRAS* mutations were found in 50% of CRC patients. Comparison between mutated and wild type *KRAS* tumors revealed a trend toward right-sided tumours (30% and 4.3% vs 16% and 1.1%, p-value = 0.032, respectively) and peritoneal metastases (34% vs 19%, p-value = 0.014) (Table 1).

**Table 1:** Association between KRAS mutations and baseline clinicopathological characteristics of the study subjects (n=194)

KRAS				
		WILD (%)	MUTATED (%)	p-Value
Age	>70	14 (14.4)	20 (20.6)	
	61-70	34 (35.1)	30 (30.9)	
	51-60	30 (30.9)	21 (21.6)	0.120
	41-50	14 (14.4)	12 (12.4)	
	<40	5 (5.2)	14 (14.4)	
Gender	Male	48 (49.5)	57 (58.8)	0.195
	Female	49 (50.5)	40 (41.2)	
Site	Right*	14 (15.7)	28 (30.1)	
	Appendiceal	1(1.1)	4 (4.3)	0.032
	Left	38 (42.7)	37 (39.78)	
	Rectum	36 (40.44)	24 (25.80)	
Type	Adenocarcinoma	88 (100)	92 (97.87)	0.388
	Neuroendocrine	0 (0)	1 (1.06)	
	other	0 (0)	1 (1.06)	
Grade	Low grade	64 (79.01)	74 (84.09)	0.394
	High grade	17 (13.77)	14 (15.90)	
Metastasis	Liver	51 (53.1)	60 (61.85)	0.220
	Lung	30 (30.92)	43 (44.32)	0.054
	Peritoneum*	18 (18.56)	33 (34.02)	0.014
	Other	29 (29.89)	27 (27.83)	0.751

Independent predictors of *KRAS* mutations were age and the presence of lung and peritoneal metastases (Table 2).

**Table 2:** Independent predictors of KRAS mutations: multivariable logistic regression model OR: odds ratio; CI: confidence interval

	OR (95% CI)	P-value
KRAS		
Age/years	0.980 (0.955 – 0.999)	0.048
Sex (Female)	0.881 (0.957 – 1.004)	0.689
Lung Metastasis	1.838 (1.007 – 3.405)	0.046
Peritoneal Metastasis	2.304 (1.157 – 4.586)	0.046

Correlation of *KRAS* mutations with overall and progression-free survival

**Figure 1:**
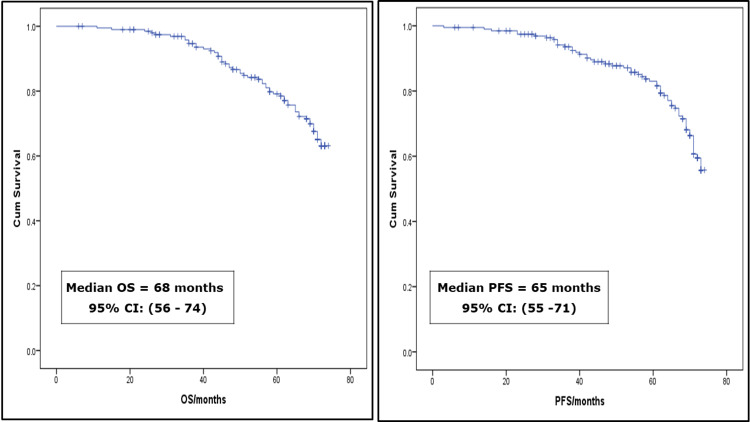
Median overall survival (OS) and progression-free survival (PFS) of the study participants

The median overall survival (OS) was 68 months (95% confidence interval 56-74), whereas the median progression-free survival (PFS) was 65 months (95% confidence interval 55-71), across all stages (Figure 1).

Cox hazard regression analysis revealed that older age at diagnosis, gender (females), tumor grade (high), microsatellite instability (MSI), tumor stage (the primary tumor), and the presence of distant metastasis were independent prognostic factors for poor OS. There was no significant difference between *KRAS-*mutated tumors and wild-type *KRAS* tumors for the hazard of mortality (hazard ratio (HR): 0.653, 95% confidence interval 0.873 - 1.134, p = 0.131) (Table 3).

**Table 3:** Cox hazard regression of the independent survival predictors for overall survival (OS) HR, hazard ratio; CI, confidence interval; MSI, microsatellite stability; TNM, tumor-node-metastasis

	P-value	HR	95.0% CI	
			Lower	Upper
Age (years)	< 0.001	1.041	1.019	1.064
Sex (Female)	0.029	0.537	0.307	0.939
Grade (High)	0.044	2.283	1.035	3.940
MSI	0.036	1.685	1.039	2.734
TNM Stage (T)	0.001	1.015	1.009	1.021
Distant Metastasis	0.007	2.043	1.210	3.448
*KRAS* (Mutated)	0.131	0.653	0.873	1.134

Adjusted multivariate analysis revealed that older age at presentation, the presence of MSI, tumor nodal stage (N), and the presence of liver and lung metastasis, and recurrence were poor prognostic factors for PFS. There was no significant difference between mutated and wild *KRAS* tumors for the hazard of progression (HR ratio: 0.756, 95% confidence interval 0.229 - 2.497, p = 0.646) (Table 4).

**Table 4:** Cox hazard regression of the independent survival predictors for progression-free survival (PFS) MSI, microsatellite stability; TNM, tumor-node-metastasis

	P-value	HR	95.0% CI	
			Lower	Upper
Age (years)	0.017	1.026	1.005	1.047
Sex (Female)	0.158	0.658	0.405	1.158
MSI	0.044	2.567	1.021	7.284
TNM Stage (N)	0.001	1.012	1.006	1.017
Liver Metastasis	0.047	1.754	1.009	3.167
Lung Metastasis	0.026	1.808	1.074	3.043
Recurrence	0.003	8.395	2.049	14.397
*KRAS* (Mutated)	0.646	0.756	0.229	2.497

## Discussion

The present study shows that *KRAS* mutations were found in 50% of the studied CRC patients. Despite that, this proportion was higher than those reported in western countries and the Asian region (35-40%) [12-13]. This is in accordance with the mutation rates reported in Saudi Arabia [8,10].

In the current study, the age and gender of the patients did not significantly affect the mutation status. This finding was also observed in previous studies of *KRAS* mutations in Saudi Arabia [8-10]. This could be explained on the basis of a low number of study participants (n=51) in the study by Mulla et al. [9].

With regard to age and gender, some studies have observed that *KRAS* mutations occur more frequently in women and younger patients [14]. However, others have shown that *KRAS* mutation rates are higher in patients older than 50 years of age versus those younger than 50 years of age [15]. It seems that these differences could be related to geographical differences, sampling techniques, and the size of studied participants [16].

Characteristically, the present study has shown that, compared to wild-type *KRAS* tumors, *KRAS*-mutated ones showed a trend toward right-sided tumors and peritoneal metastases. This finding could have a significant clinical impact and is in agreement with that of the meta-analysis carried out by Xie et al. who collected data from 17 studies with 11,385 colon cancer patients and observed that *KRAS* mutation was more frequent in right-sided than left-sided colon cancers [17]. The authors supported their observation by the fact that the right and left sides of the colon have different embryologic origins. Thus, tumors that originate from the two sites of the colon have different molecular carcinogenic characteristics, including KRAS, BRAF mutations, and microsatellite instability (MSI) [18-19].

Our survival analyses revealed interesting results. Despite the fact that OS and PFS were significantly related to different clinicopathologic variables of the study cohort, they were not affected by the *KRAS* mutational status.

Studies of *KRAS* mutations among Saudi patients could have similar epidemiologic characteristics [8-10]. However, comparing the current study findings with those of previous studies is interesting. While we observed significant impacts of different clinicopathological variables on the survival of our cohorts, this finding was observed by Alharbi et al. [8] and not observed by Mulla et al. and Zekri et al. [9-10]. Similar to our findings, Alharbi et al. [8] and Zekri et al. [10], *KRAS* mutation status did not impact CRC patients’ survival.

It is to be noted that *KRAS* mutation is not the deterministic carcinogenic factor for CRC but acts in combination with other carcinogenic and clinicopathologic factors such as patient sex, age, consistent molecular subtypes, and tumor staging. However, other scientists believe that the mutation rate of the *KRAS* gene is not related to such clinicopathologic factors as gender, age, degree of differentiation, tumor location, and type of specimen [20]. The existence of such differences and dilemmas may be related to many factors such as dietary habits, geographical location, and sample size.

Taking into consideration the fact that our data were extracted from a tertiary hospital with better patient care and multidisciplinary teams and had enrolled a relatively good number of CRC patients who were followed for survival for six years, our results have important clinical implications.

Limitations

The limitations of the present study include the inherited features of being a retrospective study that was carried out at a single center.

## Conclusions

The results of the current study show that the prevalence of *KRAS *mutations in CRC patients is similar to that observed in previous studies of Saudi patients. *KRAS* mutations showed a trend toward right-sided tumors and peritoneal metastases. Survival was significantly related to different clinicopathologic variables of the study cohort like older age at diagnosis, tumor stage, and the presence of distant metastasis. However, both overall and progression-free survival were not affected by the *KRAS* mutational status. Further, larger, and/or multicenter studies are needed.
